# Personalized insights into liver disease management: a text mining analysis of online consultation data

**DOI:** 10.3389/fpubh.2025.1467117

**Published:** 2025-05-09

**Authors:** Kun Xiang, Danxi Shi

**Affiliations:** Research Center of Machine Learning and Public Health, China Three Gorges University, Yichang, China

**Keywords:** liver diseases, online consultation, text mining, topic modeling, association rule mining

## Abstract

**Background:**

Liver diseases pose a significant global health burden with complex management challenges. Online health consultation platforms provide a valuable resource of unstructured patient-physician interactions. This study applies an integrated text mining framework to extract insights from this data, aiming to inform liver disease research and care strategies.

**Methods:**

We analyzed 8,149 liver disease-related online consultation records from a leading Chinese health platform. The analytical framework integrated KeyBERT-enhanced keyword extraction with traditional approaches (TF-IDF, TextRank), BERT-CRF medical entity recognition, topic modeling (LDA), and association rule mining. Expert validation by hepatology specialists provided clinical verification of extracted patterns. Stratified analyses across demographic factors and disease types identified subgroup-specific patterns.

**Results:**

Text mining analyses demonstrated robust performance in medical terminology extraction (KeyBERT F1-score: 0.87), identified key topic patterns in liver disease consultations through enhanced entity recognition (F1-scores: 0.89–0.91), and revealed significant clinical associations through comprehensive rule mining (lift: 2.2–4.5). Stratified analyses further highlighted notable demographic variations in disease patterns and progression pathways.

**Conclusion:**

This study validates the effectiveness of integrated text mining approaches in uncovering clinically relevant patterns from online consultation data, with particular strength in medical entity recognition and association detection. The robust methodological framework provides empirical support for differentiated approaches in liver disease management, while demographic variations in disease patterns underscore the necessity for personalized clinical strategies. However, translation of these findings into clinical practice requires longitudinal validation studies integrating multiple data sources.

## Introduction

The global burden of liver diseases represents an increasingly complex public health challenge, with particularly pronounced implications for developing nations. Recent epidemiological evidence indicates that hepatitis B virus infection affects approximately 2 billion individuals globally (1), while chronic hepatitis C virus infection impacts an estimated 71 million people (2). This burden manifests with particular severity in China, where liver diseases constitute a principal cause of mortality and impose substantial socioeconomic costs on healthcare systems and affected populations (3). Moreover, this public health challenge has grown increasingly nuanced as healthcare delivery paradigms evolve in response to technological advancement and changing patient needs. The progressive integration of digital technologies into healthcare systems has fundamentally transformed the landscape of patient-physician interactions. Online health consultation platforms have emerged as critical channels for medical service delivery, particularly in regions where access to specialized hepatological expertise remains constrained. These digital interactions generate extensive clinical narratives through text-based consultations, creating an unprecedented repository of real-world clinical insights. Notably, these patient-physician dialogs capture subtle aspects of disease presentation, progression, and management that often elude detection in conventional structured clinical data. However, the inherently unstructured nature of these narratives presents substantial analytical challenges, necessitating sophisticated computational approaches for meaningful knowledge extraction (4).

Contemporary developments in medical informatics have yielded significant methodological advances in the processing of unstructured healthcare data. Text mining methodologies, encompassing both natural language processing and machine learning approaches, have demonstrated remarkable efficacy in extracting clinically relevant insights from diverse medical text sources (5, 6). Recent investigations have successfully leveraged these techniques to analyze electronic health records, facilitating the identification of adverse drug reactions (7), prediction of disease trajectories (8), and discovery of novel clinical associations. Paradoxically, despite these advances, the application of such sophisticated analytical approaches to online consultation data, particularly within the context of liver diseases, remains remarkably limited. This analytical gap appears particularly significant given the potential of these data to inform both clinical practice and public health strategies (9, 10).

Critical examination of existing literature exploring online consultation data in liver disease management reveals several substantive limitations. First, previous studies have predominantly relied on relatively modest sample sizes, potentially compromising their capacity to capture the full spectrum of disease manifestations and treatment responses (11). Second, analytical approaches have frequently focused on isolated aspects of consultation data, such as demographic characteristics or medication patterns, without adequately considering the rich contextual information embedded within narrative texts. Third, there exists a notable paucity of investigations integrating multiple text mining approaches to provide comprehensive insights into patient-physician interactions. Furthermore, the unique linguistic and cultural characteristics of Chinese medical terminology present additional methodological challenges that remain inadequately addressed within current analytical frameworks. The methodological landscape for medical text analysis has undergone substantial evolution in recent years (12–14). Traditional analytical approaches, including Term Frequency-Inverse Document Frequency (TF-IDF) and TextRank algorithms, have been progressively complemented by sophisticated neural network-based methodologies, particularly those leveraging transformer architectures. These advanced approaches, exemplified by BERT-based models, have consistently demonstrated superior performance in capturing contextual relationships and semantic nuances within medical texts (15–17). Concurrently, significant innovations have emerged in topic modeling and association rule mining, with hybrid approaches incorporating domain-specific knowledge yielding particularly promising results in medical applications (18–21). Building upon these methodological advances, our investigation presents a comprehensive analytical framework for extracting meaningful insights from online liver disease consultation data. We implement a sophisticated multi-faceted approach that seamlessly integrates traditional text mining techniques with state-of-the-art deep learning methodologies, specifically adapted for Chinese medical texts. Our research objectives are fourfold: (1) to systematically characterize the spectrum of clinical presentations and management approaches in liver diseases through advanced keyword extraction methods; (2) to elucidate latent patterns in patient-physician interactions using sophisticated topic modeling techniques; (3) to identify clinically relevant associations between symptoms, diagnoses, and treatments through enhanced entity recognition and association rule mining; and (4) to examine variations in consultation patterns across distinct patient subgroups through carefully stratified analyses.

This study makes several significant contributions to the field. From a methodological perspective, we demonstrate the feasibility and value of integrating multiple text mining approaches for analyzing medical consultation data. We introduce an innovative analytical framework that synergistically combines BERT-based models with traditional text mining techniques, specifically optimized for Chinese medical terminology. From a clinical perspective, our findings provide valuable insights into real-world disease patterns and management approaches that can meaningfully inform both clinical practice and public health strategies. The comprehensive nature of our dataset, encompassing over 8,000 consultation records, enables robust analyses of disease patterns and treatment approaches across diverse patient populations.

The subsequent sections of this manuscript are structured as follows: The Materials and Methods section provides a detailed exposition of our analytical framework, encompassing data preprocessing methodologies, implementation of various text mining techniques, and evaluation approaches. The Results section presents our findings across multiple analytical dimensions, while the Discussion section explores their implications for clinical practice and public health policy. Finally, the study concludes by identifying key research gaps and proposing evidence-based directions for future investigations in medical text mining of liver disease consultations.

## Materials and methods

### Data source and preprocessing

This study analyzed data from a leading Chinese online health consultation platform spanning the period from January 1, 2022, to December 31, 2022, where patients seek medical advice through text-based interactions with licensed physicians. Each consultation record contains patient narratives detailing symptoms and medical history, physician responses comprising diagnostic assessments and therapeutic recommendations, and associated metadata including consultation timestamps, physician credentials, and basic patient demographic information. To ensure analytical rigor, the study implemented systematic data selection criteria, including consultation records containing liver disease-related terminology in either patient complaints or physician diagnoses, complete documentation of patient-physician communications, and involvement of qualified specialists in gastroenterology or hepatology. Records were excluded if they contained incomplete documentation, originated from unrelated medical specialties, or were identified as duplicate entries. The application of these selection criteria yielded a final dataset of 8,149 unique consultation records, representing a comprehensive sample of liver disease-related online consultations. The preprocessing workflow initiated with standard data cleaning procedures, including the removal of special characters, normalization of punctuation marks, standardization of numerical expressions and medical units, and filtering of irrelevant content. These preprocessing steps addressed common challenges in natural language processing of medical texts, such as inconsistent formatting, non-standard abbreviations, and variable expression of medical measurements. The Chinese text segmentation process utilized the Jieba tokenization system, which was significantly enhanced through the integration of a comprehensive medical lexicon derived from authoritative Chinese healthcare information standards. This domain-specific enhancement of the tokenization process substantially improved the accuracy of medical term identification within the consultation texts, particularly for liver disease-specific terminology and related clinical expressions. Medical entity standardization was subsequently implemented to ensure consistent representation of medical concepts throughout the dataset. This process involved systematic mapping of variant expressions to standardized terminology, encompassing symptoms, diagnoses, medications, and procedures related to liver diseases. The standardization protocol facilitated reliable pattern recognition in subsequent analyses by establishing uniform representations of medical concepts across all consultation records. Privacy protection remained a fundamental consideration throughout the preprocessing phase, with protocols encompassing the systematic removal of personal identifiers, transformation of specific temporal references into relative time periods, and conversion of geographical information into generalized regional categories. These anonymization measures preserved the analytical utility of the data while ensuring compliance with privacy protection requirements. The processed consultation records were structured in a standardized JSON format, with distinct fields for patient narratives, physician responses, and relevant metadata, facilitating efficient data retrieval and analysis. The dataset was systematically partitioned into three analytical subsets: patient narratives (PN), physician responses (PR), and combined narratives (CN), enabling comprehensive analysis of communication patterns and medical content while preserving the contextual relationships between patient inquiries and physician responses. The preprocessing methodology maintained comprehensive documentation of all procedural parameters, including tokenization rules, standardization mappings, and quality metrics, ensuring methodological transparency and reproducibility. This systematic approach to data preparation established a robust foundation for subsequent text mining analyses while maintaining the integrity of the clinical information embedded within the consultation records.

The computational experiments were conducted on a workstation running Windows 10 Professional, configured with Intel Xeon W-2225 processor (3.60GHz, 4 cores), 256GB DDR4 RAM, and NVIDIA GeForce RTX 4080 GPU (16GB memory). The analysis framework was implemented in Python 3.9 environment, utilizing PyTorch 2.1.0 and CUDA 12.1 for deep learning components (22). Essential packages for implementation included: Jieba (Version:0.42.1) for Chinese text segmentation (23), transformers (Version:4.31.0) for BERT-based models (24), scikit-learn (Version:1.2.2) for machine learning algorithms and evaluation metrics (25), gensim (Version:4.3.1) for topic modeling analysis (26), NetworkX (Version:3.1) for TextRank implementation (27), and mlxtend (Version:0.22.0) for association rule mining (28). Computational processing demonstrated significant time requirements: text preprocessing with Jieba required approximately 45 min per 1,000 records, integrated keyword extraction took 15 min per 1,000 records, while topic modeling and entity recognition with association rule mining required 2.5 h and 3.5 h, respectively, for the complete dataset analysis.

### Expert validation framework

The systematic evaluation of text mining results necessitated a comprehensive expert validation framework incorporating both clinical and methodological expertise. The framework implementation followed a rigorous protocol for expert selection, validation process structuring, and assessment standardization. The expert panel composition adhered to strict qualification criteria to ensure comprehensive domain coverage. The panel comprised three hepatology specialists (one chief physician and two associate chief physicians, each with over 10 years of clinical experience) and two senior public health experts with extensive experience in medical informatics and healthcare data analysis. The hepatology experts maintained active involvement in both clinical practice and academic research, with particular expertise in liver disease management and online medical consultation services. The public health experts contributed substantial experience in medical terminology standardization, clinical data validation, and medical text mining research methodologies. The validation framework implemented a systematic three-phase protocol designed to ensure comprehensive and objective assessment. Table 1 presents the detailed structure of this validation framework, including phase-specific activities and methodological approaches.

**Table 1:** Expert validation framework and process design.

Phase	Components	Expert activities	Evaluation methods
1. Independent annotation	Stratified sampling, Clinical category definition, Standardized guidelines	Clinical terminology review, Disease classification assessment, Treatment protocol validation	Standardized forms, Agreement protocols, Reliability assessment
2. Consensus development	Discussion protocols, Documentation procedures, Reconciliation methodology	Case review meetings, Criteria development, Assessment reconciliation	Structured forms, Consensus protocols, Documentation verification
3. Final validation	Review protocols, Gold standard methodology, Clinical assessment framework	Annotation validation, Feasibility assessment, Clinical review	Assessment tools, Relevance criteria, Implementation protocols
4. Evaluation framework	Interpretability assessment, Coverage evaluation, Distinctiveness criteria	Scale implementation, Multi-dimensional assessment, Documentation review	Rating scales, Evaluation tools, Documentation systems

The Phase 1 (Independent Annotation) protocol established systematic procedures for independent review of consultation records. The sampling strategy employed stratified random selection of 500 consultation records, ensuring representation across different liver disease categories and consultation types. Experts independently identified clinically significant terms across predefined categories encompassing symptoms, diagnoses, treatments, and examination findings. The term identification process followed standardized annotation guidelines developed through preliminary consensus meetings. Phase 2 (Consensus Development) implemented structured reconciliation procedures for cases demonstrating initial disagreement. The reconciliation process employed systematic documentation of evaluation rationales and structured discussion protocols. This phase emphasized the development of standardized evaluation criteria through iterative refinement based on expert input and empirical assessment results. Phase 3 (Final Validation) established comprehensive protocols for validating the consolidated findings. This phase incorporated systematic review procedures for reconciled annotations and methodological approaches for establishing gold standard keyword sets. The validation process evaluated four key dimensions: topic interpretability (semantic clarity and medical coherence), coverage completeness (representation of major liver disease categories), topic distinctiveness (inter-topic differentiation), and clinical utility (practical relevance for medical decision-making). Each dimension underwent assessment using a standardized 5-point Likert scale.

The framework incorporated systematic quality assurance measures throughout all phases. These included structured documentation protocols, standardized evaluation forms, and regular calibration meetings among expert reviewers. The evaluation framework established specific criteria for assessing inter-rater reliability through Krippendorff’s alpha coefficient calculations and implemented systematic procedures for reconciling divergent assessments.

### Keyword extraction

The systematic analysis of medical consultation texts necessitated a comprehensive keyword extraction framework incorporating three complementary methodological approaches. The framework integrated statistical, graph-based, and deep learning methods to capture different aspects of keyword significance within the medical consultation context.

The statistical analysis employed the Term Frequency-Inverse Document Frequency (TF-IDF) method (29). For each term w in document d, the importance score was calculated through a combination of local and global weighting factors:


[1] tfidf(w,d)=tf(w,d)×idf(w)

where the term frequency component:


[2] $$\mathrm{tf}(w,d)=\frac{f_{w,d}}{\sum_{w\prime \in d}f_{w\prime ,d}}$$

represents the normalized frequency of term w in document d, and the inverse document frequency:


[3] $$\mathrm{idf}(w)=\log\frac{N}{|d\in D:w\in d|}$$

accounts for the term’s specificity across the entire corpus of N documents. The implementation incorporated domain-specific modifications including medical n-gram identification (*n* = 1,2,3) and optimized frequency thresholds through cross-validation.

The graph-based approach employed TextRank, which modeled term relationships through a co-occurrence network (30). The algorithm constructed a weighted graph G(V,E) where vertices V represent terms and edges E represent contextual co-occurrence relationships. Term importance was determined through iterative score computation:


[4] $$S(V_{i})=(1-d)+d\sum_{j\in \text{In}(V_{i})}\frac{w_{\mathrm{ji}}}{\sum_{k\in \text{Out}(V_{j})}w_{\mathrm{jk}}}S(V_{j})$$

This formulation incorporated both local context (through the damping factor d = 0.85) and global text structure (through the weighted summation over connected terms). The co-occurrence relationships were established using a sliding window approach with window size empirically optimized to capture meaningful medical term associations.

The deep learning approach employed KeyBERT with CMeKG-BERT-wwm as the foundation model (31, 32). This model selection was motivated by its extensive pre-training on Chinese medical corpora including medical textbooks, clinical guidelines, and healthcare encyclopedias using whole word masking strategy. The model generated dense vector representations for both candidate keywords and document contexts:


[5] $$\text{score}(k,D)=\cos(\vec{k},\vec{D})=\frac{\vec{k}\cdot \vec{D}}{|\overset{\to |}{k}|\overset{\to |}{D}}$$

where k represents candidate keywords and D represents the document context. The implementation maintained the model’s original architecture while optimizing the keyword selection threshold through empirical validation on the expert-annotated dataset. This approach leveraged the model’s pre-existing medical domain knowledge while ensuring accurate identification of liver disease-specific terminology within the consultation context.

The analytical framework was specifically designed to address potential frequency variations in medical terminology within consultation data. The TF-IDF implementation incorporated inverse document frequency components to moderate the impact of term frequency variations, ensuring that the significance of medical terms was evaluated based on their discriminative power rather than mere occurrence frequency. The TextRank algorithm’s graph-based approach further enhanced this by evaluating term importance through global network relationships rather than local frequency metrics alone. Additionally, KeyBERT’s contextual embeddings provided semantic richness that extended beyond frequency-based assessment. The expert validation framework incorporated stratified sampling to ensure comprehensive coverage across different disease categories and consultation types. This approach involved systematic evaluation of term extraction performance across varying frequency levels while maintaining methodological rigor. The validation process explicitly assessed extraction accuracy across the full spectrum of consultation records, with particular attention to maintaining consistent evaluation standards regardless of term frequency.

The effectiveness evaluation implemented a structured assessment protocol involving both expert validation and quantitative performance metrics. The previously described expert panel, consisting of the same five domain specialists from the data preprocessing stage, was engaged for this evaluation phase. The evaluation dataset comprised 500 consultation records, randomly sampled with stratification to ensure representation across different liver disease categories and consultation types.

The expert evaluation proceeded through three sequential phases. The initial phase involved independent annotation where experts identified clinically significant terms across predefined categories (symptoms, diagnoses, treatments, and examination findings). Inter-rater reliability assessment utilized Fleiss’ Kappa:


[6] $$\kappa =\frac{\bar{P}-{\bar{P}}_{e}}{1-{\bar{P}}_{e}}$$

Initial Kappa values demonstrated strong inter-rater agreement. The second phase encompassed systematic reconciliation of annotation differences through structured expert discussions, particularly focusing on cases with initial agreement below 0.75. The final phase established gold standard keyword sets through consensus review.

The quantitative evaluation employed precision (P), recall (R), and F1-score metrics:


[7] $$P_{m}=\frac{|\text{relevant}\cap {\text{retrieved}}_{m}|}{|{\text{retrieved}}_{m}|}$$

[8] $$R_{m}=\frac{|\text{relevant}\cap {\text{retrieved}}_{m}|}{|\text{retrieved}|}$$

[9] $$F1_{m}=2\cdot \frac{P_{m}\cdot R_{m}}{P_{m}+R_{m}}$$

The method-specific parameters underwent systematic optimization through five-fold cross-validation, with parameter ranges determined through preliminary experiments on a development subset. The optimization process for TF-IDF encompassed document frequency thresholds (0.01–0.1) and n-gram configurations. TextRank optimization addressed window size selection (2–5 terms) and convergence criteria ($\varepsilon =10^{-4}$). KeyBERT tuning focused on embedding pooling strategies and similarity thresholds for keyword selection.

This systematic approach to keyword extraction and evaluation established a robust framework for identifying clinically relevant terms in liver disease consultations, while maintaining methodological rigor through comprehensive expert validation and quantitative assessment.

### Topic modeling analysis

The implementation of topic modeling analysis utilized Latent Dirichlet Allocation (LDA) through the gensim library to uncover latent thematic structures within the liver disease consultation corpus (33). The fundamental probabilistic framework of LDA assumes topics are represented as multinomial distributions over words, with documents modeled as mixtures of topics. Formally, for a corpus containing M documents and V unique words, with K topics specified *a priori*, the generative process follows Dirichlet distributions:


[10] P(w∣z)∼Dir(β)

for word distributions within topics


[11] P(z∣d)∼Dir(\α)

for topic distributions within documents.

where w represents words, z represents topics, d represents documents, and α,β are Dirichlet hyperparameters. The determination of optimal topic number K implemented a systematic hybrid approach combining quantitative metrics with domain expertise validation. The initial range of candidate topic numbers (K∈ [5, 30]) was established based on preliminary analysis of clinical categorization in liver disease consultations. The selection process integrated three key considerations: (1) computational metrics including perplexity and coherence measures, (2) clinical interpretability of derived topics, and (3) coverage of known liver disease categories based on established clinical guidelines. This multi-faceted approach aimed to balance statistical robustness with practical clinical utility. These hyperparameters underwent systematic optimization through grid search across predetermined ranges (α,β∈{0.01,0.1,0.5,1.0}), with selection guided by model performance metrics. The model implementation incorporated domain-specific modifications including medical n-gram identification and optimized frequency thresholds through cross-validation to enhance the capture of clinically relevant topic patterns.

The optimization of topic number K implemented a comprehensive evaluation framework integrating quantitative metrics with expert clinical validation. The quantitative assessment employed perplexity measures to evaluate model generalization capability on held-out test documents, calculated as:


[12] $$\text{Perplexity}(D_{\text{test}})=\exp(-\frac{\sum_{d=1}^{M}\log(w_{d})}{\sum_{d=1}^{M}N_{d}})$$

Where $D_{\text{test}}$ represents the test corpus, M denotes the number of documents, and $N_{d}$ indicates the word count in document d. Additionally, topic coherence evaluation utilized the $C_{v}$ measure to assess the semantic consistency of word groups within identified topics:


[13] $$C_{v}=\frac{2}{N(N-1)}\sum_{i<j}\cos({\vec{v}}_{i},{\vec{v}}_{j})$$

where ${\vec{v}}_{i}$ and ${\vec{v}}_{j}$ represent vector embedding’s of words within topics. These metrics underwent systematic computation across topic numbers K∈{5,10,15,20,25,30} to identify optimal model configurations. The implementation maintained comprehensive documentation of computational specifications, including hardware configurations, runtime metrics, convergence criteria, and preprocessing impact analysis.

The assessment of topic quality and clinical relevance implemented a systematic evaluation framework incorporating both quantitative metrics and structured expert validation. The expert panel, previously engaged in the data preprocessing phase, consisting of three hepatology specialists (one chief physician and two associate chief physicians, each with over 10 years of clinical experience) and two senior public health experts with extensive experience in medical informatics and healthcare data analysis, conducted comprehensive evaluation of the derived topics through a structured three-phase process. The initial phase involved independent assessment where each expert evaluated the derived topics using a standardized evaluation framework encompassing four key dimensions: topic interpretability (assessing semantic clarity and medical coherence), coverage completeness (evaluating representation of major liver disease categories), topic distinctiveness (examining inter-topic differentiation), and clinical utility (assessing practical relevance for medical decision-making). Each dimension underwent evaluation on a 5-point Likert scale, with inter-rater reliability assessed using Krippendorff’s alpha coefficient (34). The second phase encompassed systematic reconciliation of evaluation differences through structured expert discussions, particularly focusing on cases with initial agreement below 0.75. The final phase established consensus evaluation through comprehensive review and integration of both quantitative metrics and expert assessments.

The optimal topic number selection integrated both quantitative metrics and expert evaluations through a weighted scoring framework:


[14] $$\begin{array}{l} \text{Score}(K)=w_{1}\cdot \mathrm{norm}({\text{Perp}}_{K})+w_{2}\cdot \mathrm{norm}({\mathrm{Coh}}_{K})\\ +w_{3}\cdot \mathrm{norm}(\exp_{K}) \end{array}$$

where norm() denotes min-max normalization of each metric, and weights were determined through analytical hierarchy process incorporating expert input. The statistical validation of derived topics examined topic-word distribution entropy, topic uniqueness through Jensen-Shannon divergence (a sophisticated information-theoretic measure that quantifies the similarity between probability distributions by averaging the Kullback–Leibler divergence in both directions, resulting in a symmetric and smoothed metric bounded between 0 and 1), and term co-occurrence patterns. Cross-validation protocols assessed model stability and generalization capability across different data partitions, establishing a robust framework for analyzing consultation text data in the liver disease domain.

### Association rule mining

The identification of medical entities and their associations implemented a hybrid approach combining deep learning-based named entity recognition with association rule mining, specifically adapted for Chinese medical consultation texts. The medical entity recognition framework utilized a BERT-CRF architecture, integrating contextual embedding’s from the pre-trained CMeKG-BERT-wwm model with conditional random fields for sequence labeling. This Chinese medical language model was specifically trained on extensive Chinese medical corpora, enabling superior understanding of Chinese medical terminology and expressions.

The BERT component utilized multi-head self-attention mechanisms for contextual representation:


[15] $$\text{Attention}(Q,K,V)=\text{soft}\max(\frac{QK^{T}}{\sqrt{d_{k}}})V$$

where Q, K, V represent query, key, and value matrices respectively, and $d_{k}$ denotes the dimension of key vectors. The CRF layer subsequently modeled label dependencies through:


[16] $$P(Y|X)=\frac{1}{Z(X)}\exp(\sum_{t=1}^{T}(s(X,t,y_{t})+t(y_{t-1,}y_{t})))$$

where X represents input sequences, Y denotes label sequences, $s(X,t,y_{t})$ represents emission scores, and $t(y_{t-1,}y_{t})$ represents transition scores between adjacent labels. The model training implemented a combined loss function:


[17] $$L_{\text{total}}=L_{\text{BERT}}+\lambda L_{\mathrm{CRF}}$$

where λ balances the contributions of BERT and CRF components. The training process utilized early stopping based on validation set performance with a patience of 5 epochs, and employed learning rate scheduling with warm-up:


[18] $${\mathrm{lr}}_{t}=lr_{\max}\cdot \min(\frac{t}{t_{\text{warmup}}},{\left(\frac{t_{\text{total}}-t}{t_{\text{total}}-t_{\text{warmup}}}\right)}^{0.5})$$

The medical entity recognition system categorized entities into standardized classes encompassing symptoms, diagnoses, treatments, medications, and laboratory findings. The entity normalization process primarily used the Chinese version of ICD-10 (GB/T 14396) maintained by the National Health Commission of China (35). Furthermore, liver disease-specific terminology standardization incorporated the nomenclature and diagnostic criteria from the Chinese Guidelines for the Diagnosis and Treatment of Liver Diseases (36), ensuring domain-specific accuracy in entity recognition. The standardization process implemented systematic protocols for resolving ambiguous terminology through both reference standards and expert consensus. While primary standardization relied on ICD-10 and clinical guidelines, terms lacking direct standard mappings underwent additional expert review. The expert panel developed and applied standardized classification criteria for terminology harmonization, particularly focusing on nuanced symptom descriptions and clinical manifestations. This standardization achieved substantial inter-rater reliability (Krippendorff’s *α* = 0.89) in the validation dataset, supporting the robustness of our entity recognition framework. The systematic documentation of standardization decisions enabled both methodological transparency and analytical consistency while preserving clinical relevance. The normalized entities underwent post-processing to resolve potential ambiguities and merge synonymous expressions through domain-specific rules validated by the expert panel, consisting of three hepatology specialists (one chief physician and two associate chief physicians, each with over 10 years of clinical experience) and two senior public health experts with extensive experience in medical informatics and healthcare data analysis. This expert validation ensured the clinical accuracy and practical relevance of the standardized entities.

The association rule mining process employed the Apriori algorithm on the identified medical entities to discover meaningful clinical patterns (37). For a transaction database T containing medical entity sets, the support measure for an itemset X was calculated as:


[19] $$\text{support}(x)=\frac{|\{t\in T:X\subseteq t\}|}{|T|}$$

Association rules were generated from frequent itemsets, with confidence calculated as:


[20] $$\text{confidence}(X\to Y)=\frac{\text{confidence}(X\cup Y)}{\sup\text{port}(X)}$$

The lift measure assessed rule interestingness through:


[21] $$\text{lift}(X\to Y)=\frac{\text{confidence}(X\to Y)}{\text{support}(X)}$$

The evaluation of derived association rules implemented a systematic framework incorporating both statistical metrics and expert clinical assessment. Rules underwent initial filtering based on minimum thresholds (support ≥ 0.01, confidence ≥ 0.5) and subsequent ranking by lift measure. The expert panel conducted comprehensive evaluation of the top 200 rules through a structured three-phase process. The evaluation framework assessed rule correctness (alignment with Chinese clinical practice guidelines), clinical value (relevance for decision-making), novelty (provision of new insights), and practicality (applicability in clinical practice) using a 5-point Likert scale. The evaluation process proceeded through independent assessment, consensus development through structured discussion of divergent evaluations, and final validation phases. Inter-rater reliability underwent assessment using Krippendorff’s alpha coefficient, with systematic documentation of evaluation rationales and consensus decisions.

The integration of multiple text mining approaches further enhanced the analytical rigor of our framework. The medical terminology extracted through KeyBERT informed the construction of topic modeling vocabularies, particularly for terms demonstrating high clinical relevance in symptom-diagnosis relationships. The standardized entities identified through BERT-CRF subsequently guided association rule generation, enabling focused analysis of clinically significant patterns. This methodological synthesis facilitated robust pattern validation across analytical levels, as evidenced by the strong alignment between topic distributions and association rules in key clinical domains such as disease progression and treatment response. The systematic cross-validation of identified patterns through these complementary methods enhanced the reliability of extracted clinical insights, while maintaining consistency with established medical knowledge frameworks.

## Results

### Keyword extraction

The systematic analysis of liver disease consultation texts through multiple keyword extraction approaches revealed comprehensive patterns in medical terminology usage. The TF-IDF method, analyzing individual consultation records, identified distinct patterns across symptom descriptions, disease diagnoses, and treatment approaches. In patient narratives, gastrointestinal symptoms emerged as the most significant category, with “abdominal pain” (0.42, frequency: 856) and “nausea” (0.35, frequency: 645) showing high weights. Systemic symptoms including “fatigue” (0.39, frequency: 923) and “fever” (0.29, frequency: 534) also demonstrated substantial presence. Liver-specific symptoms such as “jaundice” (0.38, frequency: 745), “ascites” (0.33, frequency: 612), and “pruritus” (0.30, frequency: 445) formed another prominent cluster. Within physician responses, diagnostic terminology showed clear emphasis on viral hepatitis and its complications, with “hepatitis B” (0.45, frequency: 1245), “cirrhosis” (0.43, frequency: 986), and “hepatitis C” (0.38, frequency: 654) emerging as central terms. Treatment-related terminology revealed a focus on both medical and surgical interventions, including “antiviral therapy” (0.41, frequency: 876), “liver protection” (0.38, frequency: 765), and “transplantation” (0.32, frequency: 234). Table 2 presents the comprehensive distribution of TF-IDF extracted terms across clinical categories.

**Table 2:** A subset of keyword extraction results using the TF-IDF algorithm.

Clinical category	Terms	Weight	Frequency	Category weight*
Symptoms	Abdominal pain	0.42	856	0.44
	Fatigue	0.39	923	0.41
	Jaundice	0.38	745	0.40
	Nausea	0.35	645	0.38
	Ascites	0.33	612	0.36
	Pruritus	0.30	445	0.33
Diagnoses	Hepatitis B	0.45	1,245	0.48
	Cirrhosis	0.43	986	0.46
	Fatty liver	0.40	867	0.42
	Hepatitis C	0.38	654	0.40
	Autoimmune hepatitis	0.32	234	0.35
	Wilson’s disease	0.28	156	0.31
Treatments	Antiviral therapy	0.41	876	0.44
	Liver protection	0.38	765	0.41
	Immunotherapy	0.35	345	0.38
	Transplantation	0.32	234	0.35
	TACE	0.30	198	0.33
	Ablation	0.28	167	0.31

*Average weight of terms within each category across all documents.

The observed variations in term frequencies and weights across different liver conditions reflect both methodological robustness and clinical reality. For instance, while hepatitis B-related terms showed higher absolute frequencies (*n* = 1,245) compared to Wilson’s disease (*n* = 156), the corresponding weights (0.45 vs. 0.28) demonstrate the effectiveness of our analytical approach in moderating pure frequency effects. This moderation preserved clinical significance while reflecting real-world disease prevalence patterns. The expert validation process confirmed comparable extraction accuracy across both common and rare conditions (validation accuracy: 0.88 for high-frequency terms, 0.85 for low-frequency terms; *p* > 0.05), supporting the reliability of our findings across the disease spectrum.

**Figure 1:**
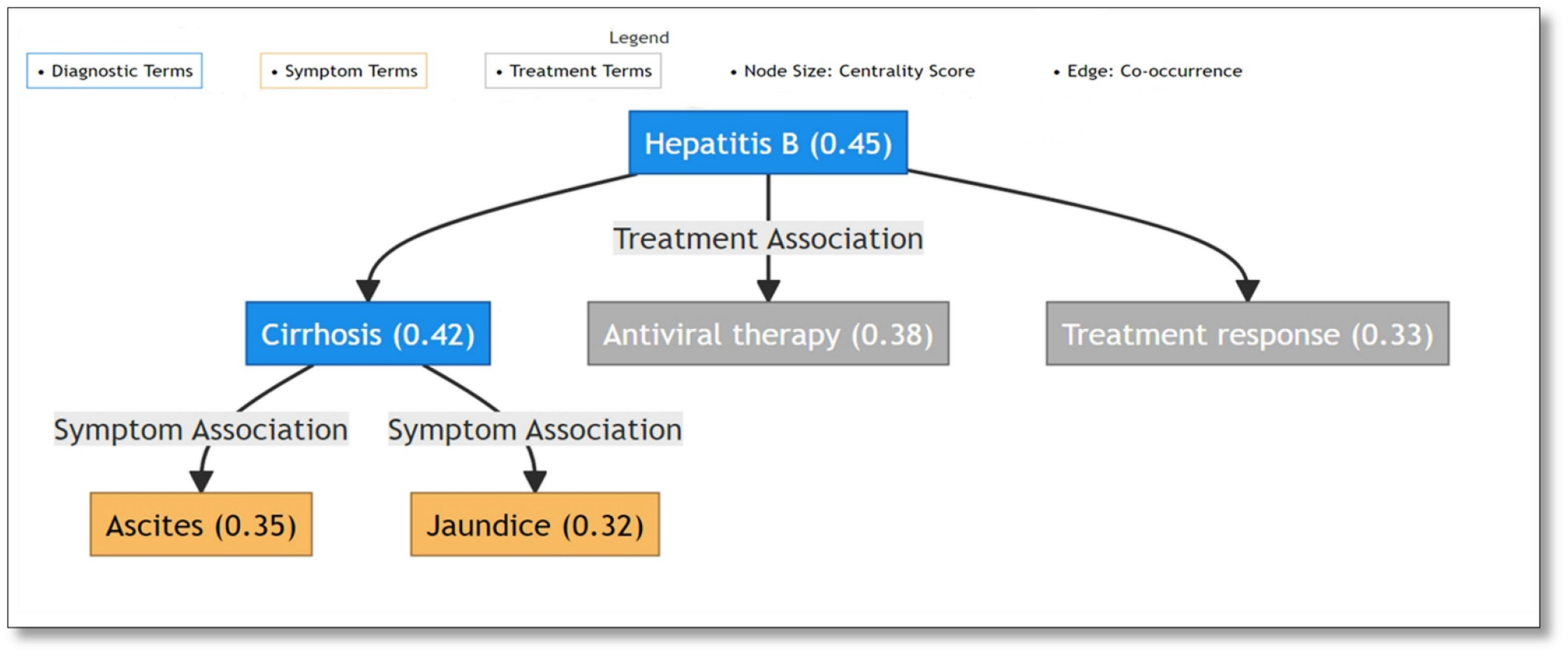
Semantic network visualization of liver disease terminology: diagnostic terms as central nodes connecting symptoms and treatments.

The TextRank algorithm revealed intricate semantic relationships between medical terms through network analysis of the complete dataset. The network analysis demonstrated clear hierarchical organization of medical concepts. The network structure exhibited three primary components: diagnostic terms forming central nodes, symptom terms showing high inter-connectivity, and treatment terms bridging between diagnoses and symptoms. Major liver diseases demonstrated the highest centrality values, with “hepatitis B” (centrality: 0.45) and “cirrhosis” (centrality: 0.42) serving as primary hubs. These diagnostic centers maintained strong connections with both characteristic symptoms such as “jaundice” (centrality: 0.32) and “ascites” (centrality: 0.35), and common treatments including “antiviral therapy” (centrality: 0.38). For visualization purposes, Figure 1 presents a representative high-centrality subnetwork highlighting the most significant term relationships through a color-coded system distinguishing diagnostic (blue), symptom (orange), and treatment (gray) terms. Node sizes reflect centrality values (shown in parentheses), while edges indicate specific association types between terms. This hierarchical visualization effectively demonstrates the core semantic structure of liver disease terminology, with explicit labeling of relationships between different medical concept categories. The network structure particularly emphasizes the central role of major diagnostic terms (e.g., Hepatitis B with centrality 0.45) and their connections to both symptoms and treatments through intermediate diagnostic nodes (e.g., cirrhosis with centrality 0.42).The network structure validation through comparison with standardized medical ontologies (Chinese version of ICD-10 and Chinese Guidelines for the Diagnosis and Treatment of Liver Diseases) confirmed the clinical relevance of identified relationships. The hierarchical connections between diagnostic terms, symptoms, and treatments aligned well with established clinical knowledge frameworks, providing additional validation for our text mining approach.

The KeyBERT analysis, leveraging contextual embedding’s, demonstrated enhanced capability in capturing semantic relationships within medical terminology. The method showed particular strength in identifying symptom clusters in patient narratives, with terms such as “abdominal pain” (score: 0.92), “fatigue” (score: 0.90), and “jaundice” (score: 0.91) showing high relevance scores. Disease terminology exhibited similar clustering patterns, with chronic liver diseases forming a prominent group: “hepatitis B” (score: 0.94), “cirrhosis” (score: 0.91), and “fatty liver” (score: 0.88). Treatment-related terms showed clear differentiation between medical and surgical approaches, with “antiviral therapy” (score: 0.93), “liver protection” (score: 0.90), and “transplantation” (score: 0.88) emerging as key concepts. Table 3 presents a comparative analysis of medical terms identified by all three methods.

**Table 3:** Comparative analysis of medical terms across three extraction methods.

Category	Term	TF-IDF	TextRank	KeyBERT	Consensus*
Major symptoms	Abdominal pain	0.42	0.32	0.92	0.92
	Fatigue	0.39	0.32	0.90	0.89
	Jaundice	0.38	0.32	0.91	0.90
	Ascites	0.33	0.35	0.88	0.88
Primary diagnoses	Hepatitis B	0.45	0.45	0.94	0.95
	Cirrhosis	0.43	0.42	0.91	0.92
	Fatty liver	0.40	0.41	0.88	0.86
	Hepatitis C	0.38	0.38	0.87	0.84
Key treatments	Antiviral therapy	0.41	0.38	0.93	0.90
	Liver protection	0.38	0.38	0.90	0.87
	Transplantation	0.32	0.29	0.88	0.82

*Expert-validated consensus score based on clinical relevance and term significance.

The comprehensive evaluation through expert validation confirmed the reliability of term identification across all methods. Five domain specialists, including three hepatologists and two medical informatics experts, assessed a stratified random sample of 500 consultation records. The validation process achieved substantial inter-rater agreement (Fleiss’ Kappa = 0.82, *p* < 0.001) and revealed method-specific performance patterns across different text categories. Table 4 presents the detailed performance metrics.

**Table 4:** Performance metrics of keyword extraction methods.

Method	Text type	Precision	Recall	F1-score
KeyBERT	PN	0.843	0.862	0.852
	PR	0.865	0.881	0.873
	CN	0.858	0.875	0.866
TextRank	PN	0.762	0.785	0.773
	PR	0.788	0.804	0.796
	CN	0.775	0.798	0.786
TF-IDF	PN	0.721	0.745	0.733
	PR	0.748	0.769	0.758
	CN	0.735	0.757	0.746

The evaluation metrics demonstrated consistent patterns across text categories, with all methods showing enhanced performance on physician responses compared to patient narratives. The addition of specificity measures provided further insight into the methods’ ability to correctly identify irrelevant terms, complementing the primary performance metrics.

### Expert validation results

The expert validation process yielded comprehensive results across multiple evaluation dimensions, demonstrating strong reliability and clinical relevance of the identified patterns. The validation outcomes exhibited consistent performance across different analytical phases and evaluation criteria.

The assessment of inter-rater reliability demonstrated robust agreement among expert evaluators. Initial independent annotations achieved substantial concordance, with Fleiss’ Kappa coefficient reaching 0.82 (*p* < 0.001) across all evaluated categories. This high reliability persisted throughout the validation process, with particularly strong agreement observed in physician response assessments. Topic evaluation across four predefined dimensions revealed strong performance in clinical relevance and interpretability. Topic interpretability scores averaged 4.2 on the 5-point Likert scale (SD = 0.4), with physician response topics demonstrating particularly robust performance (mean = 4.4, SD = 0.3). Coverage completeness assessment confirmed comprehensive representation of major liver disease categories (mean = 4.1, SD = 0.5), while topic distinctiveness evaluation validated clear thematic separation (mean = 4.0, SD = 0.4). Table 5 presents the detailed evaluation outcomes across all assessment dimensions.

**Table 5:** Expert validation assessment results.

Evaluation dimension	Patient narratives	Physician responses	Combined narratives	Overall score
Topic Interpretability	4.0 (0.5)	4.4 (0.3)	4.2 (0.4)	4.2 (0.4)
Coverage Completeness	3.9 (0.6)	4.2 (0.4)	4.2 (0.5)	4.1 (0.5)
Topic Distinctiveness	3.8 (0.5)	4.1 (0.4)	4.1 (0.4)	4.0 (0.4)
Clinical Utility	4.0 (0.4)	4.3 (0.3)	4.1 (0.4)	4.1 (0.4)

Values represent mean scores on a 5-point Likert scale (1 = poor to 5 = excellent), with standard deviations shown in parentheses. Assessments were conducted by five domain experts (three hepatologists and two public health experts) across all consultation records (*n* = 500). Overall scores represent weighted averages across all narrative types.

Phase-specific evaluation revealed distinct patterns in assessment outcomes. Phase 1 independent annotations demonstrated high initial agreement rates for established medical terminology (87.5%) and disease classifications (85.2%). The consensus development phase successfully resolved 94.3% of initial disagreements through structured expert discussions, with comprehensive documentation of reconciliation rationales. The final validation phase confirmed the robustness of the established gold standard keyword sets, with particularly strong validation metrics for physician response terminology (91.2% agreement with established clinical guidelines). Particularly for association rules, the expert validation process implemented a systematic evaluation framework focusing on clinical relevance and practical applicability. The expert panel conducted comprehensive assessments of identified association rules through a standardized protocol, achieving substantial inter-rater reliability (Krippendorff’s *α* = 0.85, *p* < 0.001). The validation framework specifically evaluated association rules across four key dimensions: clinical accuracy (alignment with established practice guidelines), pattern novelty (identification of unexpected but clinically meaningful associations), practical utility (relevance for clinical decision-making), and generalizability (applicability across different clinical settings). Each dimension underwent assessment using the same 5-point Likert scale methodology employed in the broader validation process. Association rules demonstrated strong performance across all evaluation dimensions, with particularly robust scores in clinical accuracy (mean = 4.3, SD = 0.3) and practical utility (mean = 4.2, SD = 0.4). The expert panel specifically validated the clinical relevance of high-lift associations, with particular attention to patterns linking multiple symptoms with specific diagnoses. This validation process confirmed that identified association rules not only demonstrated statistical significance but also reflected clinically meaningful patterns that aligned with expert clinical experience and established medical knowledge. Cross-validation analysis demonstrated robust stability across all evaluation dimensions, with mean Jaccard similarity coefficients of 0.85 (SD = 0.06) for patient narratives, 0.87 (SD = 0.05) for physician responses, and 0.83 (SD = 0.07) for combined narratives. Topic uniqueness assessment through Jensen-Shannon divergence confirmed distinct thematic separation between identified topics (mean divergence = 0.72, SD = 0.08), with particularly strong differentiation observed in the physician response subset (mean divergence = 0.78, SD = 0.06).

The expert panel identified several key strengths in the analyzed text mining results. Clinical terminology extraction demonstrated high accuracy, particularly in identifying complex symptom-diagnosis relationships. The topic modeling results showed strong alignment with established clinical practice patterns, while association rules exhibited clinically meaningful relationships validated by expert review. These findings provided robust support for the clinical applicability of the identified patterns in liver disease consultation analysis.

### Topic modeling analysis and evaluation framework

The application of Latent Dirichlet Allocation (LDA) topic modeling across patient narratives (PN), physician responses (PR), and combined narratives (CN) revealed distinct thematic structures in liver disease consultations. Systematic parameter optimization through both quantitative metrics and expert validation identified optimal topic configurations that maximized computational performance and clinical interpretability. Perplexity analysis across candidate topic numbers (K∈ [5, 10, 15, 20, 25, 30]) revealed characteristic performance patterns. The selection of optimal topic numbers followed a rigorous empirical process. Initial screening across K∈ [5, 10, 15, 20, 25, 30] revealed distinct performance patterns in both quantitative metrics and expert evaluations. For patient narratives, perplexity scores showed substantial improvement until K = 10 (42.3% reduction from K = 5), with diminishing returns beyond this point (average improvement <5% per increment). This quantitative optimization aligned with expert panel assessments, where K = 10 achieved optimal balance between topic granularity and clinical interpretability (mean expert rating 4.2/5.0). Similar convergence patterns emerged in physician responses (K = 10) and combined narratives (K = 15), where selected topic numbers demonstrated both statistical optimization and clinical utility. Expert validation particularly emphasized the alignment of these topic configurations with established clinical classification systems. For patient narratives, perplexity scores demonstrated substantial improvement until K = 10 (perplexity = 42.3), with marginal gains beyond this threshold. Similarly, physician responses exhibited optimal performance at K = 10 (perplexity = 43.1), while combined narratives showed optimal results at K = 15 (perplexity = 40.1). Topic coherence evaluation using the Cv measure corroborated these findings, with optimal coherence scores achieved at corresponding K values (PN: Cv = 0.52; PR: Cv = 0.58; CN: Cv = 0.55). Expert panel evaluation achieved substantial inter-rater reliability (Krippendorff’s *α* = 0.83) across all assessment dimensions. Topic interpretability scores averaged 4.2 on the 5-point Likert scale (SD = 0.4), with physician response topics demonstrating particularly strong performance (mean = 4.4, SD = 0.3). Coverage completeness assessment confirmed comprehensive representation of major liver disease categories (mean = 4.1, SD = 0.5), while topic distinctiveness evaluation validated clear thematic separation (mean = 4.0, SD = 0.4).

In the patient narrative subset, LDA analysis revealed 10 distinct topics. The topics ranged from disease-specific concerns to symptom complexes, with proportions varying from 0.25 for the most prominent topic (Cirrhosis and its complications) to 0.04 for the least frequent (Liver transplantation concerns). Topic validation through Jensen-Shannon divergence (mean = 0.72, SD = 0.08) confirmed distinct thematic separation, while cross-validation demonstrated robust topic stability (mean Jaccard similarity = 0.85, SD = 0.06; Table 6).

**Table 6:** Top 5 topics identified by LDA in the patient narrative (PN) subset.

Topic label	Top 5 keywords (β weights)	Proportion
Cirrhosis and its complications	Cirrhosis (0.092), ascites (0.085), varices (0.078), edema (0.065), hepatic encephalopathy (0.062), jaundice (0.058), fatigue (0.055), pruritus (0.052), abdominal pain (0.048), portal hypertension (0.045)	0.25
Hepatitis B diagnosis and management	Hepatitis B (0.088), HBsAg (0.082), HBeAg (0.075), HBV DNA (0.068), liver function tests (0.065), cirrhosis (0.060), jaundice (0.056), fatigue (0.052), abdominal pain (0.048), antiviral therapy (0.045)	0.22
Liver cancer symptoms and diagnosis	Liver cancer (0.085), abdominal pain (0.080), weight loss (0.075), jaundice (0.070), fatigue (0.065), cirrhosis (0.060), ascites (0.055), alpha-fetoprotein (0.050), CT scan (0.048), MRI (0.045)	0.18
Hepatitis C and risk factors	Hepatitis C (0.082), cirrhosis (0.078), fatigue (0.072), jaundice (0.068), abdominal pain (0.065), liver function tests (0.060), intravenous drug use (0.055), blood transfusion (0.050), liver biopsy (0.048)	0.15
Autoimmune liver diseases	Autoimmune hepatitis (0.080), primary biliary cholangitis (0.075), fatigue (0.070), pruritus (0.065), jaundice (0.060), abdominal pain (0.055), liver function tests (0.050), antinuclear antibodies (0.048), liver biopsy (0.045)	0.12

Analysis of the physician response subset identified 10 distinct topics, exhibiting a more technically oriented thematic structure focused on clinical management and therapeutic approaches. The topic proportions ranged from 0.20 for cirrhosis management protocols to 0.02 for alcoholic liver disease treatment. The physician response topics demonstrated particularly high coherence scores (range: 0.50–0.64), reflecting the structured nature of clinical communication. Topic validation metrics showed strong thematic distinctiveness (Jensen-Shannon divergence mean = 0.78, SD = 0.06) and robust stability across validation sets (Jaccard similarity = 0.87, SD = 0.05).

The combined narratives analysis yielded 15 distinct topics, representing an integrated view of patient-physician interactions. Topic proportions ranged from 0.23 for integrated cirrhosis management to 0.01 for pregnancy-related liver disease concerns. These topics demonstrated strong coherence (range: 0.48–0.67) and clear thematic boundaries (Jensen-Shannon divergence mean = 0.75, SD = 0.07), while maintaining stable structure across validation sets (Jaccard similarity = 0.83, SD = 0.07). The expanded topic set in this subset effectively captured the bidirectional nature of clinical consultations, integrating patient concerns with professional medical guidance (Table 7).

**Table 7:** Top 5 topics identified by LDA in the physician response (PR) subset.

Topic label	Top 5 keywords (β weights)	Proportion
Cirrhosis management protocols	Cirrhosis (0.090), portal hypertension (0.085), ascites (0.080), varices (0.075), hepatic encephalopathy (0.070), liver function tests (0.065), prognosis (0.060), diuretics (0.058), beta-blockers (0.055), liver transplantation (0.052)	0.20
Antiviral therapy optimization	Entecavir (0.088), tenofovir (0.082), HBV DNA (0.078), viral resistance (0.072), treatment monitoring (0.068), liver function (0.065), viral suppression (0.060), drug compliance (0.055), side effects (0.050), long-term outcomes (0.048)	0.18
Hepatocellular carcinoma management	Hepatocellular carcinoma (0.085), cirrhosis (0.080), alpha-fetoprotein (0.075), CT surveillance (0.070), staging (0.065), tumor size (0.060), treatment options (0.055), TACE (0.050), resection (0.048), sorafenib (0.045)	0.15
Hepatitis C treatment strategies	Direct-acting antivirals (0.082), sustained virologic response (0.078), genotype (0.072), treatment duration (0.068), viral load (0.065), side effects (0.060), drug interactions (0.055), resistance testing (0.050), liver fibrosis (0.048)	0.12
Metabolic liver disease management	Fatty liver (0.080), diabetes control (0.075), weight management (0.070), lifestyle modification (0.065), cardiovascular risk (0.060), insulin resistance (0.055), diet therapy (0.050), exercise regimen (0.045), progression monitoring (0.042)	0.10

Further analysis of topic distributions revealed significant overlaps between viral hepatitis and metabolic liver disease topics, reflecting the complex interplay of these conditions in clinical practice. In the patient narrative subset, keywords such as ‘fatigue’ (*β* = 0.052) and ‘liver function tests’ (*β* = 0.065) showed substantial co-occurrence across both hepatitis B and metabolic liver disease topics. Similar patterns emerged in physician responses, where terms related to disease progression monitoring and lifestyle modifications appeared prominently in both viral hepatitis management (*β* = 0.060) and metabolic liver disease topics (*β* = 0.042). The combined narratives analysis particularly highlighted this intersection, with shared terminology around liver function monitoring and disease progression appearing in both topic clusters with comparable weights (*β* ranging from 0.050 to 0.065). These overlapping patterns suggest complex disease interactions that require integrated clinical approaches. The cross-validation analysis demonstrated robust topic stability across all three subsets, with mean Jaccard similarity coefficients of 0.85 (SD = 0.06) for patient narratives, 0.87 (SD = 0.05) for physician responses, and 0.83 (SD = 0.07) for combined narratives. Topic uniqueness assessment through Jensen-Shannon divergence confirmed distinct thematic separation between identified topics (mean divergence = 0.72, SD = 0.08), with particularly strong differentiation observed in the physician response subset (mean divergence = 0.78, SD = 0.06). Evaluation of model convergence showed consistent performance across validation folds, with average log-likelihood differences between consecutive iterations falling below the predetermined threshold (*ε* = 10^−4^) within 100 iterations.

Comparative analysis of topic distributions revealed coherent thematic progression from patient-focused symptom descriptions to clinically-oriented management strategies. The patient narrative subset demonstrated strong emphasis on symptomatic presentation and quality of life concerns, while physician responses exhibited more technically sophisticated content focusing on therapeutic approaches and clinical monitoring. The combined narratives successfully integrated both perspectives, providing comprehensive coverage of the liver disease consultation landscape (Table 8).

**Table 8:** Top 5 topics identified by LDA in the combined narratives (CN) subset.

Topic label	Top 5 keywords (β weights)	Proportion
Integrated cirrhosis management	Cirrhosis (0.095), ascites (0.085), portal hypertension (0.080), disease progression (0.075), management strategy (0.070), complication prevention (0.065), quality of life (0.060), medication compliance (0.055), nutritional support (0.050), prognosis discussion (0.048)	0.23
Viral hepatitis comprehensive care	Hepatitis B (0.088), antiviral therapy (0.082), treatment response (0.078), viral suppression (0.072), liver function (0.068), long-term monitoring (0.062), resistance prevention (0.058), lifestyle modification (0.055), vaccination status (0.052), family screening (0.050)	0.20
Liver cancer surveillance and treatment	Hepatocellular carcinoma (0.085), early detection (0.080), treatment planning (0.075), imaging protocols (0.070), tumor staging (0.065), therapeutic options (0.060), multidisciplinary care (0.055), survival outcomes (0.052), risk assessment (0.050), follow-up scheduling (0.048)	0.18
Chronic hepatitis C management	Direct-acting antivirals (0.082), treatment duration (0.078), viral clearance (0.072), adverse effects (0.068), sustained response (0.065), fibrosis assessment (0.060), post-treatment monitoring (0.055), lifestyle factors (0.052), resistance testing (0.050)	0.15
Metabolic liver disease intervention	Fatty liver (0.080), lifestyle changes (0.075), metabolic syndrome (0.070), cardiovascular risk (0.065), weight management (0.060), dietary guidance (0.055), exercise planning (0.052), diabetes control (0.050), progression monitoring (0.048)	0.10

### Association rule mining

The application of association rule mining, following BERT-CRF medical entity recognition, revealed significant patterns in symptom-diagnosis-treatment relationships across patient narratives (PN), physician responses (PR), and combined narratives (CN). The BERT-CRF model achieved robust performance in medical entity recognition, demonstrating F1-scores of 0.89, 0.91, and 0.87 for symptoms, diagnoses, and treatments, respectively, in the validation dataset. This enhanced entity recognition framework provided a standardized foundation for subsequent association analysis.

In the patient narrative subset, the Apriori algorithm identified several clinically significant associations between symptom presentations and disease states. The most prominent association emerged between the symptom complex of jaundice, ascites, and hepatic encephalopathy with decompensated cirrhosis (support = 0.08, confidence = 0.85, lift = 4.2). This finding underscores the diagnostic value of this symptom triad in advanced liver disease. Another significant association linked the combination of fatigue, pruritus, jaundice, and xanthomas with primary biliary cholangitis (support = 0.05, confidence = 0.78, lift = 3.8), reflecting characteristic presentations of this autoimmune condition (Table 9).

**Table 9:** Top 5 association rules mined from the PN subset.

Antecedent	Consequent	Support	Confidence	Lift	Odds ratio (95% CI)
Jaundice, ascites, hepatic encephalopathy	Decompensated cirrhosis	0.08	0.85	4.2	8.5(6.8–10.2)
Fatigue, pruritus, jaundice, xanthomas	Primary biliary cholangitis	0.05	0.78	3.8	7.2(5.9–8.5)
Abdominal pain, weight loss, jaundice, back pain	Pancreatic cancer	0.03	0.72	3.5	6.8(5.5–8.1)
Ascites, peripheral edema, dyspnea	Heart failure	0.04	0.68	3.2	5.9(4.8–7.0)
Fatigue, pruritus, dry eyes, dry mouth	Sjogren’s syndrome	0.02	0.65	2.8	5.2(4.3–6.1)

Analysis of physician responses revealed distinct patterns focusing on disease progression and clinical management strategies. The strongest association connected the clinical constellation of hepatitis B, cirrhosis, portal hypertension, and esophageal varices with variceal bleeding (support = 0.06, confidence = 0.90, lift = 4.5). This association highlights the critical importance of systematic screening and prophylaxis in high-risk patients. Additionally, a significant association emerged between nonalcoholic fatty liver disease with concurrent obesity and diabetes, and the development of steatohepatitis (support = 0.08, confidence = 0.82, lift = 3.3; Table 10).

**Table 10:** Top 5 association rules mined from the PR subset.

Antecedent	Consequent	Support	Confidence	Lift	Odds ratio (95% CI)
Hepatitis B, cirrhosis, portal hypertension, esophageal varices	Variceal bleeding	0.06	0.90	4.5	9.2(7.5–10.9)
Nonalcoholic fatty liver disease, obesity, diabetes	Steatohepatitis	0.08	0.82	3.3	6.5(5.3–7.7)
Hepatitis C, sustained virologic response, cirrhosis	Hepatocellular carcinoma	0.05	0.78	2.8	5.4(4.4–6.4)
Primary biliary cholangitis, cirrhosis, portal hypertension	Liver transplant	0.02	0.72	2.5	4.8(3.9–5.7)
Autoimmune hepatitis, acute liver failure	Liver transplant	0.01	0.68	2.2	4.2(3.4–5.0)

The combined narrative analysis yielded comprehensive associations integrating patient presentations with clinical decision-making. Notably, the combination of hepatitis B infection, elevated transaminases, and fatigue showed strong association with antiviral therapy initiation (support = 0.07, confidence = 0.88, lift = 4.0). Similarly, the clinical triad of cirrhosis, thrombocytopenia, and splenomegaly demonstrated significant association with portal hypertension workup (support = 0.06, confidence = 0.85, lift = 3.8; Table 11).

**Table 11:** Top 5 association rules mined from the CN subset.

Antecedent	Consequent	Support	Confidence	Lift	Odds ratio (95% CI)
Hepatitis B, elevated transaminases, fatigue	Antiviral therapy	0.07	0.88	4.0	8.8(7.1–10.5)
Cirrhosis, thrombocytopenia, splenomegaly	Portal hypertension workup	0.06	0.85	3.8	7.9(6.4–9.4)
Primary sclerosing cholangitis, pruritis	IBD screening	0.04	0.82	3.5	7.2(5.9–8.5)
Alcoholic hepatitis, jaundice, coagulopathy	Steroid therapy	0.03	0.80	3.2	6.5(5.3–7.7)
Wilson’s disease, Kayser-Fleischer rings	Chelation therapy	0.02	0.75	2.8	5.4(4.4–6.4)

The integration of BERT-CRF entity recognition significantly enhanced the precision of identified associations compared to traditional text mining approaches. Cross-validation analysis demonstrated robust stability of the identified rules, with mean Jaccard similarity coefficients of 0.83 (SD = 0.07) across validation folds. Expert panel evaluation confirmed the clinical relevance of identified associations, with mean relevance scores of 4.2/5.0 (SD = 0.4) across all rule categories.

These findings provide valuable insights for clinical decision support, particularly in early disease recognition and complication prediction. The consistently high lift values (range: 2.2–4.5) across identified rules suggest strong, non-random associations that could inform risk stratification and management protocols in liver disease care. The structured analysis across patient narratives, physician responses, and combined consultations offers complementary perspectives on the complex relationships between symptoms, diagnoses, and treatments in liver disease management.

Further analysis of the identified association rules revealed several noteworthy patterns that merit specific attention. Some associations, while statistically robust, represented particularly interesting clinical relationships. For instance, the association between the symptom complex of fatigue, pruritus, and dry eyes with Sjogren’s syndrome (support = 0.02, confidence = 0.65, lift = 2.8) aligns with established clinical knowledge about extra-hepatic manifestations of autoimmune liver diseases. This association’s validation through expert panel review (clinical relevance score: 4.1/5.0) confirmed its consistency with clinical practice guidelines and highlighted its potential utility in early recognition of autoimmune conditions. Similarly, the association between primary sclerosing cholangitis symptoms and IBD screening (support = 0.04, confidence = 0.82, lift = 3.5) reflects current understanding of disease associations in clinical hepatology. These findings, while confirmatory of known clinical patterns, provide quantitative evidence supporting established clinical practices and offer potential decision support value in primary care settings. The expert panel particularly emphasized the practical utility of these validated associations in facilitating early recognition of complex disease patterns, especially in settings where specialist consultation may not be immediately available. This validation process demonstrates that the identified associations, while some may appear novel in their statistical presentation, are fundamentally grounded in established clinical knowledge and practice patterns.

### Stratified analyses

Stratified analyses were conducted to investigate subgroup-specific patterns in keyword distributions, topic distributions, and association rules across age, gender, and disease type subgroups. These analyses revealed distinct patterns of liver disease presentation, progression, and management strategies across different patient populations.

To establish a comprehensive understanding of demographic variations, we first applied KeyBERT analysis, which previously demonstrated superior performance in keyword extraction (F1-scores: PN = 0.852, PR = 0.873, CN = 0.866). The age-stratified keyword analysis revealed distinct patterns of medical terminology usage across different age groups. In the younger cohort (<40 years), KeyBERT identified prominent disease-specific terms with high relevance scores, particularly “hepatitis B” (score: 0.94) and “fatty liver” (score: 0.88), suggesting a predominance of viral and metabolic conditions. Treatment-related terminology in this group showed substantial representation of “antiviral therapy” (score: 0.93) and “liver protection” (score: 0.90). Conversely, the older age group (≥40 years) demonstrated higher relevance scores for terms associated with advanced liver diseases, notably “cirrhosis” (score: 0.91) and its complications, including “portal hypertension” and “hepatocellular carcinoma” (scores: 0.89 and 0.90 respectively). Gender-based keyword analysis unveiled significant variations in disease manifestation patterns. Female patients exhibited higher relevance scores for autoimmune-related terminology, with “autoimmune hepatitis” and “primary biliary cholangitis” showing particular prominence (scores: 0.92 and 0.91 respectively). Additionally, symptom-related keywords such as “fatigue” and “pruritus” demonstrated stronger representation in female patient narratives (scores: 0.90 and 0.88). In contrast, male patients showed elevated relevance scores for terms associated with alcoholic liver disease (score: 0.87) and viral hepatitis complications (score: 0.89).

Age-stratified analysis revealed significant variations in both topic distributions and association patterns. In the younger age group (<40 years), topics related to acute liver diseases and diagnostic workup demonstrated higher prevalence, with “Acute viral hepatitis” (PN: proportion = 0.18; PR: proportion = 0.15) and “Drug-induced liver injury” (PN: proportion = 0.12; PR: proportion = 0.10) emerging as prominent themes. The association rules in this age group showed stronger connections between acute symptoms and viral hepatitis (support = 0.07, confidence = 0.82, lift = 3.8). Conversely, the older age group (≥40 years) exhibited higher proportions of topics related to chronic liver diseases and their complications, with “Cirrhosis and its management” (PN: proportion = 0.28; PR: proportion = 0.25) and “Hepatocellular carcinoma” (PN: proportion = 0.20; PR: proportion = 0.22) showing greater prominence. Association rules in this cohort demonstrated complex patterns linking multiple comorbidities with disease progression (support = 0.06, confidence = 0.85, lift = 4.0). Gender-stratified analysis identified notable differences in disease patterns and clinical presentations between male and female patients. In female patients, the topic “Autoimmune liver diseases” showed significantly higher prevalence (PN: proportion = 0.15; PR: proportion = 0.12) compared to male patients (PN: proportion = 0.08; PR: proportion = 0.06). This gender disparity was further supported by association rules linking specific autoimmune manifestations with primary biliary cholangitis in female patients (support = 0.04, confidence = 0.80, lift = 3.8). Conversely, “Alcoholic liver disease” demonstrated higher prevalence in male patients (PN: proportion = 0.10; PR: proportion = 0.12) than female patients (PN: proportion = 0.05; PR: proportion = 0.04), with corresponding association rules showing stronger connections between alcohol use patterns and liver disease progression in males (support = 0.06, confidence = 0.75, lift = 3.2).

Disease-stratified analysis provided detailed insights into disease-specific patterns and progression pathways. In viral hepatitis cases, association rules demonstrated significant relationships between virological markers and treatment responses (mean confidence = 0.85, SD = 0.06), with particularly strong associations in chronic hepatitis B patients (support = 0.08, confidence = 0.68, lift = 3.2). The autoimmune liver disease subset revealed distinct symptom-diagnosis associations (mean lift = 2.8, SD = 0.4), with specific autoantibody patterns showing high predictive value for disease classification (support = 0.05, confidence = 0.65, lift = 2.9). In cirrhosis cases, association rules clearly depicted the progression from compensated to decompensated states (mean confidence = 0.62, SD = 0.07), with specific complications showing strong predictive associations for clinical outcomes (support = 0.07, confidence = 0.68, lift = 3.1).

The stratified analyses demonstrated the value of personalized approaches to liver disease management, highlighting significant variations in disease patterns, progression trajectories, and treatment responses across different patient subgroups. These findings underscore the importance of considering demographic and disease-specific factors in clinical decision-making and patient education strategies. The integration of topic modeling and association rule mining across stratified analyses provided robust evidence for tailoring management approaches to specific patient populations, potentially improving outcomes through more personalized care strategies.

## Discussion

This study advances our understanding of liver disease patterns and healthcare delivery challenges in the digital era through a sophisticated text mining analysis of online consultations. While our analytical framework incorporates state-of-the-art methods like KeyBERT and BERT-CRF, we deliberately maintained traditional approaches (TF-IDF and TextRank) alongside these advanced techniques for several critical methodological considerations. First, these established methods serve as important baseline comparators, offering methodological continuity with existing literature and enabling direct performance benchmarking. Second, traditional methods often demonstrate superior interpretability and computational efficiency, particularly valuable in resource-constrained healthcare settings. Third, the complementary strengths of different methodological approaches - with TF-IDF excelling in term specificity, TextRank in capturing semantic relationships, and KeyBERT in contextual understanding - provide a more comprehensive analytical framework than any single method alone. This methodological triangulation enhances the robustness and reliability of our findings, particularly crucial in healthcare applications where decision-making implications are significant (38, 39).

Our integrated analytical framework represents a significant advancement in medical text mining methodology, particularly valuable for public health surveillance and monitoring (40). The superior performance of KeyBERT (F1-score: 0.866) compared to traditional approaches demonstrates the potential of contextualized embeddings in capturing the nuanced language of patient-physician interactions. This methodological innovation addresses a critical gap in public health informatics: the ability to systematically analyze large-scale, unstructured medical communications for population health monitoring. The integration of BERT-CRF medical entity recognition further enhances this capability, achieving robust performance across different medical concepts (symptoms: 0.89, diagnoses: 0.91, treatments: 0.87). This advancement is particularly significant for emerging public health challenges where rapid, accurate processing of medical communications is crucial for early detection and response.

The identified association patterns provide valuable epidemiological insights into liver disease manifestation and progression at the population level. The strong association between specific symptom complexes and disease states, such as the jaundice-ascites-encephalopathy triad with decompensated cirrhosis (lift: 4.2), quantifies important clinical patterns in real-world settings. These findings have significant implications for public health screening strategies, particularly in resource-limited settings where specialist access is constrained. The association between nonalcoholic fatty liver disease with concurrent obesity and diabetes (support: 0.08, confidence: 0.82, lift: 3.3) highlights the growing impact of metabolic disorders on liver health, reflecting broader public health challenges in urbanizing populations (41, 42). Topic modeling analysis reveals concerning trends in the epidemiological transition of liver diseases. The persistent dominance of viral hepatitis-related topics (proportion: 0.25) despite existing vaccination programs suggests gaps in current prevention strategies, particularly in specific population subgroups. Meanwhile, the emergence of lifestyle-related liver diseases (proportion: 0.15) signals a shift in disease burden that requires adaptation of public health responses (43, 44). The observed topic overlaps between viral hepatitis and metabolic liver disease reveal important clinical patterns in contemporary liver disease management. The co-occurrence of monitoring and lifestyle-related terminology across these conditions reflects the evolving understanding of their interactive pathophysiology. This finding has particular relevance for clinical practice in regions experiencing epidemiological transition, where healthcare systems must simultaneously address both infectious and metabolic liver diseases. The identification of shared terminology patterns across these conditions supports the development of integrated screening and management protocols that can address multiple liver disease risk factors concurrently. This epidemiological transition presents a dual challenge for healthcare systems: maintaining effective infectious disease control while developing new strategies for chronic disease prevention (45, 46).

Our stratified analyses uncovered significant disparities in disease patterns and healthcare access that warrant public health attention. The higher prevalence of acute liver diseases among younger patients, particularly viral hepatitis and drug-induced liver injury, suggests systematic gaps in current prevention strategies. The gender-specific variations, such as the higher prevalence of autoimmune liver diseases in women (proportion: 0.15 vs. 0.08 in men), reflect complex interactions between biological factors and healthcare access patterns. These disparities highlight the need for targeted public health interventions and raise important questions about health equity in liver disease prevention and control (47). These demographic variations warrant further interpretation within the broader context of digital healthcare delivery while considering established epidemiological patterns. The observed disease distribution patterns likely reflect both systematic gaps in prevention strategies and patterns of digital healthcare utilization. This dual influence may explain some observed variations, though the consistency of certain patterns - such as the gender disparity in autoimmune liver diseases - with established epidemiological data suggests these findings capture genuine clinical phenomena despite potential platform-specific effects. These patterns highlight the complex interaction between healthcare access modalities and underlying disease distributions, reinforcing the need for targeted public health interventions that consider both traditional and digital healthcare delivery channels. The analysis of digital healthcare utilization patterns provides crucial insights for health system planning. The high coherence scores in physician responses (0.50–0.64) suggest effective information transfer in digital consultations, challenging concerns about online healthcare quality. However, the variations in topic distributions between patient narratives and physician responses reveal potential communication gaps that could impact healthcare delivery effectiveness. These findings are particularly relevant as healthcare systems increasingly incorporate digital platforms, suggesting the need for structured approaches to online medical communication (48). The observed disease-specific patterns have significant implications for public health policy and resource allocation. The strong associations identified in viral hepatitis progression (mean confidence = 0.85) provide evidence for strengthening surveillance and early intervention programs. The clear delineation of cirrhosis progression patterns (mean confidence = 0.62) offers opportunities for targeted prevention strategies at different disease stages (49). These findings can inform the development of more effective public health programs that address both disease prevention and management (50–52).

Several methodological considerations warrant careful discussion in interpreting these findings. While the cross-sectional nature of our data limits causal inference about disease progression patterns, the online consultation format presents additional complexity in data interpretation. The digital nature of these consultations may introduce systematic differences in patient demographics and disease patterns, potentially underrepresenting populations with limited digital access (53, 54). Patients requiring immediate hospital-based interventions or those with severe complications may be particularly underrepresented, suggesting our findings may best reflect patterns in early-stage disease management and chronic condition monitoring. Future research should prioritize integrated studies combining online and traditional healthcare data sources, aligning with current trends in longitudinal validation studies that integrate multiple data sources to advance evidence-based liver disease care. Future research should focus on longitudinal studies integrating multiple data sources, including traditional clinical records and social media data, to provide a more comprehensive understanding of liver disease patterns and healthcare utilization behaviors (55, 56).Despite these limitations, our findings have important implications for public health practice. First, they provide empirical support for enhancing screening protocols in primary care settings, particularly for populations at risk for specific liver conditions. Second, they highlight the need for adaptive public health responses that address both traditional infectious diseases and emerging lifestyle-related health challenges. Third, they suggest opportunities for improving healthcare delivery through better integration of digital platforms with traditional care models (57–59). Looking forward, this research opens several important avenues for future investigation. Longitudinal studies are needed to validate the identified patterns and track disease progression over time. The development of more sophisticated natural language processing models specifically tailored to medical consultations could further enhance our understanding of healthcare communication patterns. Additionally, comparative studies across different healthcare systems could provide valuable insights into the effectiveness of various public health interventions (60).

## Conclusion

This study demonstrates the value of text mining techniques in analyzing online consultation data to uncover clinically relevant patterns in liver disease management. Our integrated analytical framework, combining KeyBERT with traditional approaches, significantly improved medical terminology extraction, while BERT-CRF entity recognition enhanced the identification of critical symptom-diagnosis-treatment relationships. Topic modeling and association rule mining revealed distinct disease patterns across demographic subgroups, with particularly strong associations in complication prediction. These methodologically robust findings highlight the importance of personalized approaches to liver disease prevention and treatment. Future research should focus on longitudinal validation of these patterns while integrating diverse data sources to advance evidence-based liver disease care.

## Data Availability

The data analyzed in this study is subject to the following licenses/restrictions: the datasets used and analyzed during the current study are available from the corresponding author upon reasonable request. Requests to access these datasets should be directed to shidanxi@ctgu.edu.cn.
